# The United States Supreme Court Decision in “Tooth Crowns”

**Published:** 1891-06

**Authors:** 


### THE UNITED STATES SUPREME COURT DECISION IN "TOOTH CROWNS."

THIS decision was rendered April 27 in the case of Dr. Edward S. Gaylord, of New Haven, Conn., and in opposition to the claims of the Tooth Crown Company.

It will be remembered that in 1887 a suit was brought against Dr. Gaylord in the United States Circuit Court in New Haven. The judges decided the patent invalid. The case was then appealed to the Supreme Court. It reached an argument some weeks ago, the justices finally reaffirming the decision of the Circuit Court.

This conclusion will be regarded with satisfaction by the profession. Much is due to Dr. Gaylord for his long and persistent fight, and it would seem proper that this great service should receive recognition in a form worthy the man and the work.

